# Influence of the formula on the properties of a fast dispersible fruit tablet made from mango, *Chlorella*, and cactus powder

**DOI:** 10.1002/fsn3.1330

**Published:** 2019-12-13

**Authors:** Hanying Sun, Xin Wang, Jiangyu Wang, Gengqiang Shi, Lan Chen

**Affiliations:** ^1^ Institute of Food Safety and Quality University of Shanghai for Science and Technology Shanghai China

**Keywords:** direct compression, disintegration, fruit powder, moisture content, tablets, tensile strength

## Abstract

Tableting of fruit powders is gaining popularity because of the advantages it brings in, such as ease of storage, transportation, and use, and effervescent tablets could be a good alternative to accomplish fast dissolving. The present study provides a specific effervescent tablet formulation that is appropriate for the delivery of mango, cactus, and *Chlorella* fruit powder. The direct compression method was employed. A series of disintegration time, tensile strength, and moisture content tests were performed on the different formulations at each stage. The effects of effervescent agents' ratio, fruit powder proportion, acid and alkali content, and mannitol and lactose content on tablet properties were investigated. The results indicated that the tablet properties were highly influenced by formulation, especially the ratios of effervescent agents, fruit powders, acid to alkali ratio, as well as mannitol to lactose ratio. The best performing formulation was as follows, 45% effervescent agents (citric acid monohydrate:sodium bicarbonate = 1.3:1), 35% adhesives (mannitol:lactose = 1:8), and 20% mixed fruit powders (mango:cactus:*Chlorella* fruit powders = 14:5:1). With this formula, the moisture content was 3.62% and the disintegration time was 154 s, as well as a sufficient tensile strength of 2.32 MPa. Our study presented useful findings regarding the specific effects of changing ingredient ratios on tablet strength and other properties and provided a basis for the potential of using mango, cactus and microalgae powders as novel functional ingredients for fruit powder effervescent tablets. This may be used as a basis for further research on tableting.

## Graphical abstract

This study compared the properties of fast‐dissolving tablets containing mixed fruit powder and created using the direct compression method. Based on the findings regarding the specific effects of changing ingredient ratios on tablet strength and other properties, a specific tablet formulation that is appropriate for delivery of mango, cactus, and *Chlorella* fruit powder is provided.

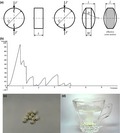

## 1 INTRODUCTION

Increased consumption of fruits has led to an increased interest in maintaining their high nutritional levels in addition to a critical eye toward their aesthetic appearance (Chemat, Zill‐e‐Huma, & Khan, 2011; Fava et al., 2011; Martínez‐Sánchez, Allende, Bennett, Ferreres, & Gil, 2006; Plaza et al., 2011; Vandekinderen et al., 2009). However, transportation, marketing, and storage of fruits are always challenging because of infections and infestations caused by microbial pathogens (Wu, Gao, Gao, Lian, Du, & Tie, 2017; Wu, Lin, et al., 2017; Zhang et al., 2015). Therefore, postharvest preservation and processing of fruit products are necessary. Fruit powder provides several advantages over fruit juices in terms of preservation, such as reduced volume, weight, and packaging requirements; easier preservation, handling, transportation, and storage; and increased shelf life (Saifullah, Yusof, Chin, & Aziz, 2016). However, because fruit powder is dry and granular in nature, it is bulky and highly hygroscopic; therefore, it is sensitive to the external environment and requires careful handling and packaging during storage and transportation.

Hence, compaction of fruit powder into tablets may be an excellent solution to these challenges. A tablet may be easily consumed as a health supplement, and this form allows ready storage, transportation, and packaging (Aziz et al., 2018). Different types of fruits have been turned into tablets such as dates and spirulina (Adiba, Salem, Nabil, & Abdelhakim, 2011), pitaya and guava (Zea, Yusof, Aziz, Ling, & Amin, 2013), and green and ripe mangoes (Ong, Yusof, Aziz, Chin, & Amin, 2014). Dissolution profile and disintegration time are key factors of fruit powder tablets when they are used in ready‐to‐serve juices and drinks. However, as the surface area of the powder is reduced when it is placed in tablet form, its hygroscopicity is reduced, and tablets take longer time to dissolve than the powdered form. The total dissolution times for pitaya, pineapple, mango, and guava powder tablets (2.5 g, diameter: 2 mm) were 55, 60, 75, and 90 min, respectively (Saifullah et al., 2016), which are not practical for consumer use. Thus, it is desirable to increase the disintegration rate of fruit powder tablets.

Effervescent tablets could be a good alternative that could overcome these drawbacks. Effervescent tablets combine the qualities of both solid and liquid dosage forms. They are dissolved or dispersed in water and consumed in a palatable liquid form while retaining the benefits of a solid dosage form such as high stability, easy portability, and accurate dose (British Pharmacopoeia, 2008). Disintegrating agents are commonly incorporated in the tablet matrix to improve the dispersibility and bio‐availability of active ingredients (Shailendra, Shailendra, Manish, Singh, & Priti, 2015). It is reported that effervescent fruit tablets completely dissolved in no more than 10 min, which is faster than the normal fruit powder tablets (Saifullah et al., 2016). Another study reported similar findings for food tablets prepared from date and spirulina powders (Adiba et al., 2011).

It should be noted that the properties of effervescent tablets are highly influenced by various factors such as the physical and chemical properties of the ingredients, the ratios of powders, and the process parameters of tableting. For instance, Saifullah et al. (2016) demonstrated that among various fruit powder tablets, pitaya and pineapple powder tablets showed the fastest dissolution (7 min), whereas guava powder tablets took the longest time (10 min) to completely dissolve. Ong et al. (2014) reported that a mixed fruit tablet containing an effervescent agent showed lower tensile strength than a mixed fruit tablet without a disintegrant.

On the other hand, as recognized functional ingredients, cactus can be used for the treatment of gastritis, fatigue, dyspnoea and liver injury following alcohol abuse(Guevara‐Figueroa et al., 2010), while microalgae is considered as a potential source of biologically active chemicals, such as proteins, fatty acids, antioxidants, vitamins and polysaccharides (Del Campo, García‐González, & Guerrero, 2007), and mango has been postulated as functional food to prevent and combat metabolic disorders, obesity‐related chronic diseases, hepatic steatosis and other comorbidities (Evans et al., 2014; Fang, Kim, Barnes, Talcott, & Mertens‐Talcott, 2018; Fang, Kim, Noratto, et al., 2018; Makare, Bodhankar, & Rangari, 2001; Natal et al., 2016). Thus, if we want to provide a ready‐to‐serve effervescent tablets with positive health benefits, the combination of mango, *Chlorella* and cactus powder could be a promising choice. And the aim of the present study was to investigate the effects of the ratios of effervescent agents, fruit powder, acid and alkali content, and mannitol and lactose content on tablet properties including disintegration time, tensile strength, and moisture content, so as to find the optimal processing parameter for this mixed fruit powder effervescent tablets.

## 2 MATERIALS AND METHODS

### 2.1 Materials

Citric acid monohydrate, sodium bicarbonate, mannitol (Shanghai FengHong), and lactose (Shanghai Changwei Pharmaceutical Accessories Technology Co. Ltd.) were obtained as samples. Ripe mango fruit powder (Kaisheng Food Co. Ltd.), *Chlorella* (*Chlorella* spp.) powder, stevia (Qingdao Famiyi Trading Co. Ltd.), cactus (*Opuntia ficus*) powder, and polyethylene glycol 6000 (Shanghai Maikelin) were obtained commercially. An FT4 Powder Rheometer (Freeman Technology), a standard thermometer (TP101 HanDian), a tray vacuum oven (Gallenkamp), a pH meter (PHS‐3B, JingKe), and a stopwatch timer (YS‐528, YiSheng) were used to measure the properties of the tablets.

### 2.2 Preparation of effervescent tablets

Flow properties and moisture ratio of all the powders were measured using the FT4 Powder Rheometer (Freeman Technology). The powders were then precisely weighted, thoroughly mixed using a Turbula shaker mixer (WAB) at 1.677*g* and for 45 min, and formulated into tablets by direct compression using a rotary tablet press (ZPS008) operating at 0.028*g*. The machine was fitted with a circular punch and dies with settings adjusted to a consistent pressure load (1.7 kN). Tablet diameter was 1 cm, and thickness was 6 mm ± 0.01. The humidity was controlled at around 45%.

### 2.3 Experimental design

#### 2.3.1 Optimization of effervescent agent concentration

Citric acid monohydrate and sodium bicarbonate were chosen as the effervescent agents and used in a ratio of 1.4:1. Mannitol and lactose were used as the adhesive agents in a ratio of 1:8. The ratio of mango powder was 20%, and 2% polyethylene glycol 6000 was added as a lubricant. The concentration of effervescent agents in tablets increased from 40% to 55% in 5% increments. Based on tablet properties of disintegration time, tensile strength, pH, and moisture content, the optimal concentration of effervescent agents in the tablet was determined.

#### 2.3.2 Optimization of fruit powder amount

After the optimal concentration of effervescent agents was determined, other parameters were held constant to determine the optimal ratio of fruit powder. The amount of *Chlorella* powder was fixed at 1%, considering its poor water solubility, and the ratio of cactus powder was decreased gradually from 8% to 0 while that of mango powder was increased from 11% to 18%. The resulting formulas were labeled A, B, C, D, E, F, G, and H. Another formulation containing 20% mango powder was used as the control and labeled I. Based on the tablet properties of disintegration time, tensile strength, pH, and moisture content, the optimal fruit powder formula for making the tablet was determined.

#### 2.3.3 Optimization of acid to alkali ratio

Using the optimal effervescent agent ratio and fruit powder formula, tablets were created to test the acid to alkali ratio. Citric acid monohydrate and sodium bicarbonate were the chosen acid and alkali, respectively, and the following ratios of citric acid monohydrate to sodium bicarbonate were examined: 1.2:1, 1.3:1, 1.4:1, 1.5:1, 1.6:1, 1.7:1, and 1.8:1. On the basis of the tablet properties of disintegration time, tablet tensile strength, pH, and moisture content, the optimal ratio of citric acid monohydrate to sodium bicarbonate in the tablet was determined.

#### 2.3.4 Optimization of mannitol to lactose ratio

To further improve tablet properties, the ratio of mannitol to lactose was adjusted. The ratios of mannitol to lactose were as follows: 1:6, 1:7, 1:8, 1:9, and 1:10. The tablet properties of disintegration time, tensile strength, pH, and moisture content were measured to determine the optimal ratio of mannitol to lactose.

#### 2.3.5 Stability studies

The stability of fast‐dissolving tablets acquired under the best performing formulation were tested according to the method reported by Jacob, Shirwaikar, and Nair (2008), that is, the tablets were stored for 20 days at 75% RH with periodic analysis (0, 10, 20 days) of tensile strength, pH, disintegration time and moisture content.

### 2.4 Measurement

#### 2.4.1 Powder properties

1. Moisture content

The moisture content was determined following standard procedures, involving drying of the powder in a tray vacuum oven at 60°C for 48 hr (Osorio‐Fierros et al., 2017).

- Flow properties

Dynamic flow properties of powders were characterized using an FT4 powder rheometer (Freeman Technology), with measurements in the dynamic module, compressibility module, and shear module. All tests were carried out in triplicate. Details of the measurement principles and operation of various modes of this device have been published previously (Freeman, 2017). Briefly, in the dynamic mode, a blade with a diameter of 23.5 mm and a helix angle of −5° traversed through a 25‐ml powder sample in a 25‐mm diameter glass vessel. The tip of the blade was maintained at a constant speed within the range of 10–100 mm/s. The energy required to move the blade through the powder during a downward traverse is defined as basic flow energy (BFE) (Rajkhowa et al., 2015).

#### 2.4.2 Tablet properties

1. Disintegration time

According to European Pharmacopoeia (2005), disintegration time is determined by allowing one tablet to disperse completely in 250 ml of purified water at room temperature. The time required for effervescence to be completed was noted using a digital stopwatch. This time was measured for six tablets, and results were presented as average ± standard deviation (Khan et al., 2014).

- Tensile strength

**Figure 1:**
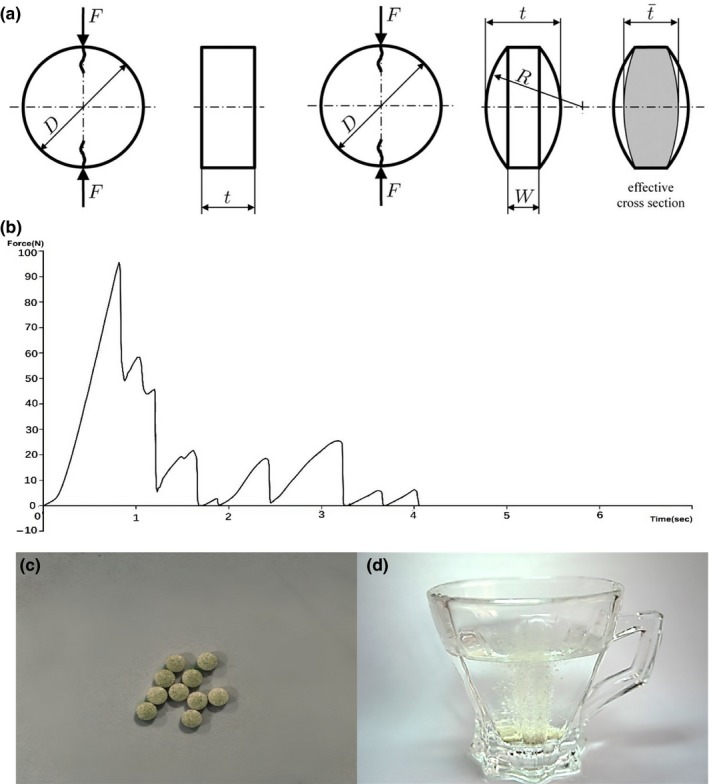
Geometry and failure behavior of tablets. (a) Schematic of a doubly convex tablet under diametrical compression. *D* (mm) is the diameter of the compact, *W* (mm) is the length of the cylindrical portion of the tablet, and it is the total thickness of the doubly convex tablet. (b) Schematic representation of breaking force of tablet containing citric acid monohydrate:sodium bicarbonate = 1.7:1. (c) Examples of the fast dispersible fruit tablets produced by direct compression. (d) The disintegration circumstances of the fast dispersible fruit tablets

Pharmaceutical tablets are fabricated by pressing powders into various shapes. It is important for tablets to have sufficient strength to endure postcompaction loading, including coating, packaging, handling, and storage. Tablet strength is thus an important quality that is tested during production. Low tensile strength can cause the tablet to break easily (Razavi, Gonzalez, & Cuitiño, 2015). We tested breaking force using a texture analyzer (TA.XT Plus), carrying out measurements for each compaction point on three sample tablets per formulation. Results were expressed as the mean value ± standard deviation. Podczeck et al. suggested that exactly the same equation can be used to calculate tensile strength in both flat and convex tablets (Podczeck, Drake, & Newton, 2013). The breaking force of a cylindrical tablet can be related to its tensile strength by Equation (1), that is, Hertz solution (Razavi et al., 2018):

(1) $$\sigma_{1}=\frac{2F}{\pi D\bar{t}}$$

where $\sigma_{1}$ is the tensile strength, *F* is the breaking force, *D* is the diameter of the tablet, and $\bar{t}$ is its thickness as shown in Figure 1a. The above expression is valid for flat cylindrical tablets that fail in tension across the symmetry plane of the loaded diameter. Breaking force was applied to tablets containing a citric acid monohydrate:sodium bicarbonate ratio of 1.7:1 (Figure 1b). The value at the first peak in Figure 1b is the diametral force necessary to break the cylindrical compact.

- pH of the solution

After disintegration time was tested, a pH meter (PHS‐3B) was used to test the pH of the solution.

- Moisture content

Moisture content in tablets is related to tablet hardness and tensile strength. The moisture content was measured after the tablets were dried in a tray vacuum oven at 60°C for 48 hr (Osorio‐Fierros et al., 2017).

### 2.5 Statistical analysis

All measurements were carried out in triplicate, except the disintegration time, which was tested with at least six samples. Statistical analysis was performed using ANOVA, and differences were considered statistically significant for *p* values <.05. Different letters in the figures designate values with a significant difference among tablets.

## 3 RESULTS AND DISCUSSION

### 3.1 Powder properties

#### 3.1.1 Powder moisture content

**Figure 2:**
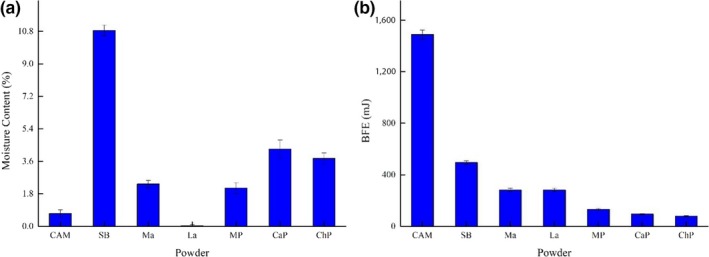
Basic material properties of fruit powders. (a) Moisture content, (b) basic flow energy (BFE). Citric acid monohydrate (CAM), sodium bicarbonate (SB), mannitol (Ma), lactose (La), mango powder (MP), cactus powder (CaP), and *Chlorella* powder (ChP)

Moisture content is a well‐known property that affects powder flowability; thus, its value has relevance for tableting procedures. A low moisture content is preferable for the preparation of effervescent tablets. Moisture content and BFE values of different ingredients are shown in Figure 2.

Sodium bicarbonate had the highest moisture content (10.8%), while lactose had the lowest value (0.03%). The moisture content of mango powder was only 2.12%, compared with 5.55% in cactus powder and 5.00% in *Chlorella* powder (Osorio‐Fierros et al., 2017), indicating that the mango powder had more hygroscopic characteristics. These values are similar to those of other fruit powders used in tablet formulations, such as pitaya (3.60%), pineapple (3.90%) (Saifullah et al., 2016), green mango (4.26%), and ripe mango (4.31%) (Ong et al., 2014).

#### 3.1.2 Powder fluidity

Good flowability and compressibility of powders is necessary for tableting (Osorio‐Fierros et al., 2017). Considering the particle size, powders that flow under gravity tend to result in higher flow energy, whereas cohesive powders show lower flow energy (Freeman, 2017). A flowable powder has higher BFE, and the BFE values correlate inversely with the Carr index. Flowable particles are less compressible, and due to more efficient packing, they have a higher bulk density and lower Carr index (Rajkhowa et al., 2015). The flow properties of the powders were determined using the FT4 Powder Rheometer.

Citric acid monohydrate had the highest BFE value, 1,488 mJ, indicating that citric acid has high fluidity, followed by sodium bicarbonate (494 mJ). The BFE of mannitol and lactose were similar, which were 282.2 and 281.4, respectively. There was little difference among the BFE values of the three fruit powders; the BFE of *Chlorella* powder was the lowest (78.8 mJ), whereas that of cactus powder was 95 mJ and that of mango powder was 132 mJ. The result was consistent with previously reported results (Osorio‐Fierros et al., 2017). There was no direct relationship between moisture content and BFE.

### 3.2 Preparation of tablets

#### 3.2.1 Effect of the ratio of effervescent agents on tablet properties

**Figure 3:**
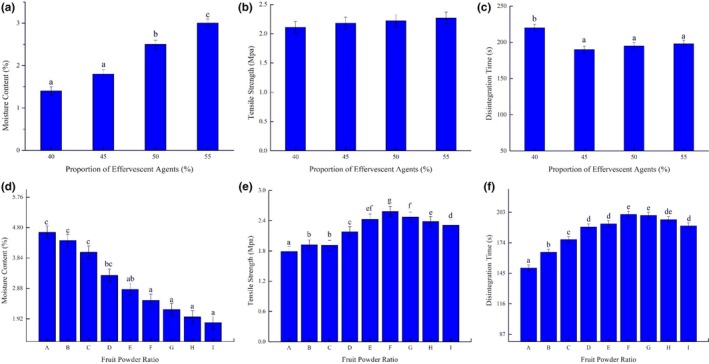
Effect of the ratio of effervescent agents (a–c) and fruit powder (d–f) on tablet properties: moisture content, tensile strength, and disintegration time. The formulations contained the following ratios of cactus:mango powder: A, 8%:11%; B, 7%:12%; C, 6%:13%; D, 5%:14%; E, 4%:16%; F, 3%:17%; G, 2%:18%; and H, 1%:19%, while I was a control formulation containing 20% mango powder. Amount of *Chlorella* powder was fixed at 1% for all formulations

As the ratio of effervescent agents in the tablet increased from 40% to 55%, moisture content, tablet tensile strength, and disintegration time also changed (Figure 3).

As shown in Figure 3a, as the ratio of effervescent agents increased, the moisture content of the tablet increased from 1.4 to 3, which caused a great deal of swelling. This effect is not desirable during the tableting process, as excessive water content can cause the tablet to be sticky. The increase in moisture content of the tablet can be explained by the higher moisture content of sodium bicarbonate.

As shown in Figure 3b, the tensile strength increased gradually from 2.11 to 2.27 MPa as the ratio of effervescent agents increased, but the difference was not significant. As the ratio of effervescent agents increased, the citric acid content increased correspondingly; thus, the hardness of the tablet and its tensile strength increased. These findings support the results reported by Olsson, Mattsson, and Nyström (1998). The tensile strength of the tablets is governed by mechanical properties, and due to their crystalline structure, citric acid particles are much bigger and harder than other fine powders. When a relatively rigid, coarse particulate material is added to a relatively ductile, fine particulate compound in the form of an ordered mixture, a fracture probably occurs around the coarse compound particles and through the inter‐particulate voids partly filled with the binder (Olsson et al., 1998).

The reaction of citric acid monohydrate with sodium bicarbonate results in the formation of carbon dioxide, which helps to break up the tablet and accelerate the dissolving process. The changes in disintegration time as a result of effervescent agent ratio are shown in Figure 3c. As the ratio of effervescent agents increased from 40% to 45%, there was a significant reduction in disintegrating time to 190 s. As the ratio of effervescent agents increased past 45%, disintegration time did not change much, with values of 195 s for 50% and 198 s for 55%. The effervescent agent produces carbon dioxide through an acid–base reaction to accelerate the dissolution of the powder in the liquid. If the acid–base reaction produces excessive bubbles, it prevents the powder from contacting water, lengthening the disintegration time. Thus, the 45% ratio of effervescent agents appears optimal.

#### 3.2.2 Effect of the ratio of mango, cactus, and *Chlorella* powders on tablet properties

The effects of different combinations of mango, cactus, and *Chlorella* powder on the tablet were compared in terms of moisture content, tensile strength, and disintegration time (Figure 3).

As the ratio of mango powder in the fruit powder increased gradually, the moisture content of the tablet decreased from 4.65% to 1.8% (Figure 3d), due to the relatively lower moisture content of mango powder compared with cactus powder. There was a significant difference in moisture content among formulations. Meanwhile, as shown in Figure 3e,f, the tensile strength increased gradually from A (1.79 MPa) to F (2.58 MPa), and the disintegration time significantly increased from A (150 s) to F (201 s), after which both tensile strength and disintegration time decreased for G, H, and I. For example, the tensile strength decreased in G, H, and I to 2.47 MPa, 2.38 MPa, and 2.31 MPa, respectively.

Tablet strength is closely associated with moisture content. Water in granules can cause multiple layers of water to form at the particle surface, which may disturb or reduce the intermolecular attraction forces and thereby reduce the tensile strength of the tablet, especially in formulations with a higher moisture content (Thapa, Lee, Choi, & Jeong, 2017). As shown in Figure 3d, formulation A had the highest moisture content, and as shown in Figure 3e, it had the lowest tensile strength. However, for formulations G, H, and I, which had the lowest water content, the water that was absorbed could increase the number of solid bridges in the surface restructuring medium, and immobile water layers absorbed at the particle surfaces could enhance particle–particle interactions. As a result, tensile strength decreased with declining water content. These results were also demonstrated by Thapa et al. (2017).

The disintegration time of formulation A was the shortest (150 s), but the tablet was very fragile, making it nonideal for practical use. Formulations B and C had similar properties. As the ratio of mango powder in the mixed fruit powder increased, the disintegration time increased accordingly for formulations A–F. For formulations G and H, the ratio exceeded 16%, and there was a slight decrease in disintegration time. The disintegration time was positively correlated with the tensile strength of the tablets. Due to the highly hygroscopic nature of cactus and *Chlorella*, these powders tend to form a gel on the surface, thus preventing water from penetrating into the tablet. In comparison, formula D, with a ratio of mango:cactus:*Chlorella* of 15:4:1, showed a relatively shorter disintegration time and lower tensile strength, so it was chosen for subsequent experiments.

#### 3.2.3 Effect of the acid to alkali ratio on tablet properties

**Figure 4:**
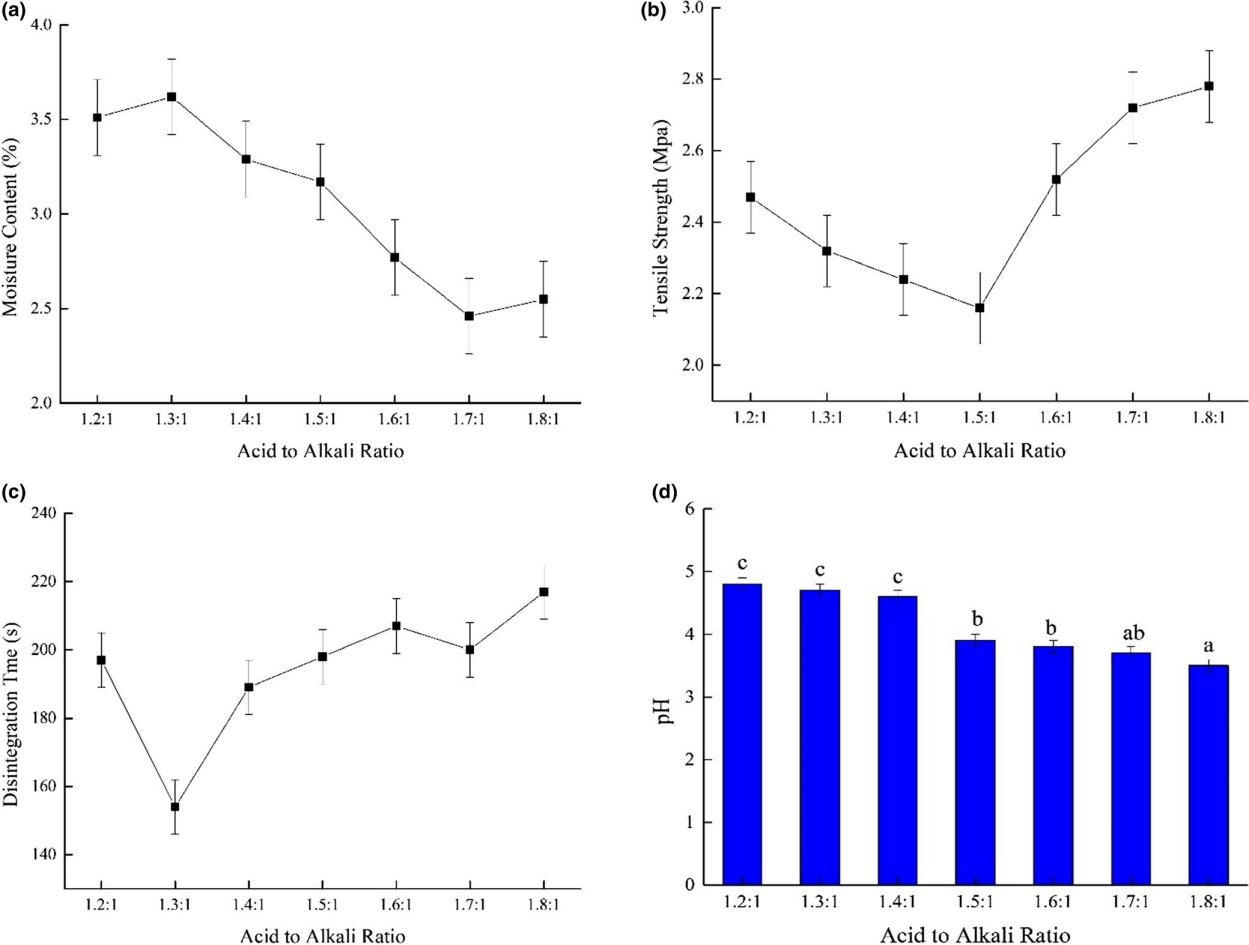
Properties of tablets containing different ratios of acid (citric acid monohydrate) to alkali (sodium bicarbonate): (a) moisture content, (b) tensile strength, (c) disintegration time, and (d) pH value

The effects of different ratios of acid to alkali, that is, citric acid monohydrate to sodium bicarbonate, were investigated. Changes in moisture content, tensile strength, disintegration time, and pH value associated with different ratios are shown in Figure 4.

The moisture content of citric acid is only 0.71%, which is much lower than that of sodium bicarbonate, 10.8% (see Figure 2a). Accordingly, as the acid ratio increased, the moisture content of the tablets declined from 3.62% to 2.46% (Figure 4a). This result was similar to that reported by Saifullah et al. (2016), who found that the moisture content values of fruit powder effervescent tablets containing pitaya, pineapple, guava, and mango were 2.87, 2.89, 3.06, and 2.71, respectively. As shown in Figure 4b, the tablet with the lowest tensile strength had an acid:alkali ratio of 1.5:1. As the ratio of citric acid to sodium bicarbonate increased from 1.2:1 to 1.5:1, the tensile strength declined gradually from 2.47 MPa to 2.16 MPa. Compared with sodium bicarbonate, citric acid has a relatively higher BFE value and high flowability (Figure 2b), making it less compressible. As the citric acid ratio increased, the porosity of the tablet increased, resulting in a decrease in tensile strength. However, when the ratio of citric acid to sodium bicarbonate increased from 1.6:1 to 1.8:1, the tensile strength increased from 2.52 MPa to 2.78 MPa. This result may be due to the fact that citric acid is a coarse particle, which could promote bonding between solid surfaces in a compact agglomerate and result in a stronger compact (Ebrahimi, Saffari, & Langrish, 2015). The physical properties of citric acid are the main factors affecting tensile strength. As a result, tensile strength showed an upward trend as the citric acid:sodium bicarbonate ratio increased past 1.5:1.

Disintegration time is normally considered the principal reference index for characterizing tablet performance. According to the European Pharmacopoeia standard for effervescent tablets, the disintegration time should be less than 5 min (Rotthäuser, Kraus, & Schmidt, 1998). As shown in Figure 4c, all the formulations disintegrated within 220 s (3.5 min), meeting the requirement. An acid–alkali ratio of 1.3:1 produced the shortest disintegrating time (154 s).

We used the fruit powder formula of mango:cactus:*Chlorella* = 15:4:1. The pH of mango juice is around 4 (Zhou et al., 2017). When the acid–alkali ratio was 1.2:1, the pH value was 4.89, as shown in Figure 4d; as the ratio of citric acid in the formulation increased, the tablet's pH value gradually decreased. For the three ratios 1.2:1, 1.3:1, and 1.4:1, the pH values were not significantly different. Thus, a pH value at the ratio of 1.3:1 was acceptable. When the acid–alkali ratio was 1.5:1, the pH value decreased significantly to 3.77. There was no significant difference between the pH values of the remaining four formulations.

#### 3.2.4 Effect of the ratio of mannitol to lactose on tablet properties

**Figure 5:**
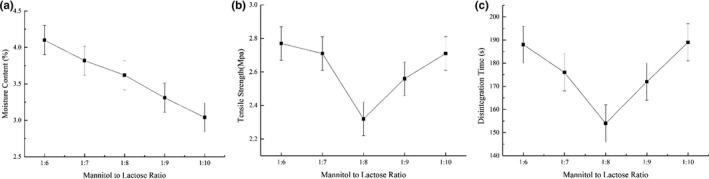
Effects of mannitol:lactose ratio on tablet properties: (a) moisture content, (b) tensile strength, and (c) disintegration time

Good flowability is an important property for direct tableting, and excipients are often used to facilitate powder flowability or to increase nonstick properties. Mannitol and lactose are brittle excipients that are frequently used as fillers and binders in direct compression formulations. The effects of the mannitol:lactose ratio on the tablet properties are shown in Figure 5.

The moisture content of the tablets declined from 4.1% to 3.04% as the ratio of mannitol to lactose decreased (Figure 5a). This is because the moisture content of lactose (0.03%) is much lower than that of mannitol (2.12%), as shown in Figure 1a. Meanwhile, as shown in Figure 5b,c, the tensile strength and the disintegration time were lowest when the ratio of mannitol to lactose was 1:8 (2.32 MPa and 154 s, respectively), while below or above this ratio, the two indexes tended to be higher. The final tablets with acceptable characters were shown in Figure 1c. Figure 1d confirmed the process of disintegration circumstances within 154 s.

These results confirm the findings of previous studies. Ziffels and Steckel (2010) stated that because of their very rough and needle‐like structure, anhydrous lactose particles exhibit a very porous structure and thus have a relatively lower tensile strength. Therefore, as the ratio of mannitol to lactose decreased from 1:6 to 1:8, the tensile strength of the tablet gradually decreased. However, when the ratio of mannitol to lactose was 1:9 and 1:10, the tensile strength increased. This result is due to the fact that plastic deformation increases with increasing amorphous content, making the bonds in the tablets stronger. Razavi, Gonzalez, and Cuitiño (2018) obtained similar results, noting that tablets with low lubrication properties showed high elastic modulus and tensile strength because they contained a large number of small particles. The small amorphous particles of lactose were able to fill up the surface irregularities as the ratio of lactose increased, so that the powder blends were closely packed. In addition, the increased hardness is thought to slow the penetration of liquid into the tablet structure, reducing the disintegrating force inside the tablet, which prolongs the disintegration process (Solaiman, Suliman, Shinde, Nas, & Elkordy, 2016).

#### 3.2.5 Stability of the effervescent tablets

Table 1 shows the stability results of the designed fast‐dissolving fruit powder tablets after storage at 75% RH for 20 days. The data indicated that the disintegration time and pH value of tablets were relatively stable, while the moisture content increased from 3.62% to 4.03%, showing significantly changes, and as a result, the tensile strength of the samples decreased from 2.32 MPa to 1.96 MPa. However, according to the European Pharmacopoeia standard for effervescent tablets (Rotthäuser et al., 1998), the disintegration time was still less than 5 min and the changes are accecptable. Meanwhile, concerning the fact that the quality of the fast‐dissolving tablets could be influenced by moisture absorption during storage, it is suggested that proper package of the fast‐dissolving tablets should be considered, so as to prolong the shelf life.

**Table 1:** Stability of effervescent tablets which stored for 0 (control) 10 and 20 days at 75% on the index of disintegration time, tensile strength, pH and moisture content

Storage time (days)	Disintegration time (s)	Tensile strength (MPa)	pH	Moisture content (%)
0	154	2.32^b^	4.7	3.62^a^
10	161	2.16^ab^	4.8	3.83^ab^
20	160	1.96^a^	4.7	4.03^b^

Different lowercase in the same column indicate significant difference in data (P < .05).

## 4 CONCLUSION

This research aimed to find the optimal formulation of the fruit powder effervescent tablets with positive health benefits. The combination of mango, *Chlorella*, and cactus powders was used as functional ingredients. The direct compression method was employed. The effects of effervescent agents' ratio, fruit powder proportion, acid and alkali content, and mannitol and lactose content on tablet properties, such as the disintegration time, tensile strength, and moisture content, were investigated. Firstly, the powder properties of the ingredients were analyzed. It turned out that citric acid has the highest fluidity, followed by sodium bicarbonate, while there was little difference among the fluidity of the three fruit powders. As the ratio of effervescent agents increased, the moisture content and tensile strength of the tablets increased gradually, while a 45% ratio of effervescent agents appears optimal for shorter disintegration. As the ratio of mango powder in the fruit powder increased gradually, the moisture content of the tablet decreased from 4.65% to 1.8%, while the tensile strength and disintegration time increased gradually. As the acid ratio increased, the moisture content of the tablets declined from 3.62% to 2.46%, while this was different from the change of tensile strength. An acid–alkali ratio of 1.3:1 produced the shortest disintegrating time (154 s). The ratio of mannitol to lactose also has an effect on the tablet properties, and the tensile strength as well as the disintegration time were lowest when the ratio of mannitol to lactose was 1:8. The stability result confirmed the changes of the tablets during storage were accecptable. Thus, the optimal formulation of the fruit powder effervescent tablets can be provided, that is, 45% effervescent agents (citric acid monohydrate:sodium bicarbonate = 1.3:1), 35% adhesives (mannitol:lactose = 1:8), and 20% mixed fruit powders (mango:cactus:*Chlorella* = 14:5:1). These results highlight the great potential of direct compression as a strategy to produce fast‐dissolving fruit powder tablets.

## CONFLICT OF INTEREST

The authors declare that they do not have any conflict of interest.

## ETHICAL APPROVAL

The protocols and procedures were ethically reviewed and approved by University of Shanghai for Science and Technology. There was no human or animal testing in this study; ethics approval and consent to participate are not applicable to this manuscript.
